# Correction: Four-dimensional analysis by high-speed holographic imaging reveals a chiral memory of sperm flagella

**DOI:** 10.1371/journal.pone.0203912

**Published:** 2018-09-07

**Authors:** The PLOS ONE Staff

## Notice of Republication

This article was republished on August 31, 2018 to correct for an error involving the Fig 1 caption that was introduced during the typesetting process. The publisher apologizes for the error. Please download this article again to view the correct version. The originally published, uncorrected article and the republished, corrected articles are provided here for reference.

## Supporting information

S1 File Originally published, uncorrected article. (PDF)

S2 File Republished, corrected article. (PDF)
