# Supplementary figures and images for: Four-dimensional analysis by high-speed holographic imaging reveals a chiral memory of sperm flagella
Source: PLoS One. 2018 Jun 28;13(6):e0199678. doi: 10.1371/journal.pone.0199678 (PMC6023239; doi:10.1371/journal.pone.0199678)

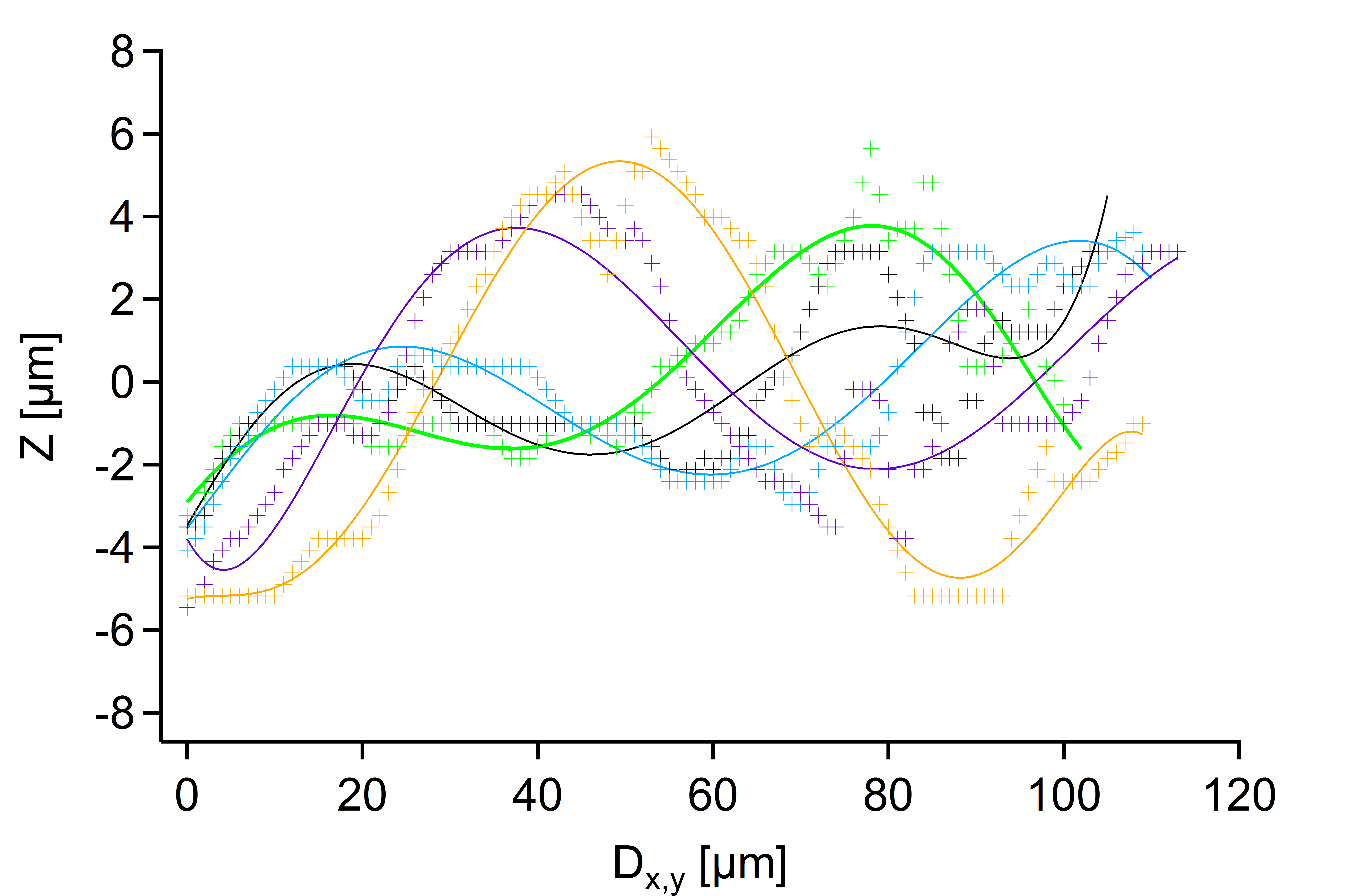

Supplement: S1 Fig — A polynomial regression proves to fit z plane excursion data adequately. The figure shows the first frames of Fig 1B as Z-plane excursions smoothed by a 7th order polynomial. According to Fig 1B, the first frame 0 is green. The data is overlapped with markers that show the unfitted data in their respective color. (TIF) [file pone.0199678.s007.tif]
