# Supplementary material for: Correction: Four-dimensional analysis by high-speed holographic imaging reveals a chiral memory of sperm flagella
Source: PLoS One. 2018 Sep 7;13(9):e0203912. doi: 10.1371/journal.pone.0203912 (PMC6128629; doi:10.1371/journal.pone.0203912)
Supplement: S1 File — (PDF) [file pone.0203912.s001.pdf]

RESEARCH ARTICLE

Four-dimensional analysis by high-speed holographic imaging reveals a chiral memory of sperm flagella

Michael Muschol, Caroline Wenders, Gunther Wennemuth*

Institute for Anatomy, University Hospital, Duisburg-Essen University, Essen, Germany

* Gunther.Wennemuth@uk-essen.de

OPEN ACCESS

Citation: Muschol M, Wenders C, Wennemuth G (2018) Four-dimensional analysis by high-speed holographic imaging reveals a chiral memory of sperm flagella. PLoS ONE 13(6): e0199678. https://doi.org/10.1371/journal.pone.0199678

Editor: Wilfried A. Kues, Friedrich-Loeffler-Institute, GERMANY

Received: February 28, 2018

Accepted: June 12, 2018

Published: June 28, 2018

Copyright: © 2018 Muschol et al. This is an open access article distributed under the terms of the Creative Commons Attribution License, which permits unrestricted use, distribution, and reproduction in any medium, provided the original author and source are credited.

Data Availability Statement: All relevant data are within the paper and its supporting information files.

Funding: Support was provided by Deutsche Forschungsgemeinschaft; WE 2344/9-1 to G.W. We also acknowledge support by the Open Access Publication Fund of the University of Duisburg-Essen.

Competing interests: The authors have declared that no competing interests exist.

Abstract

Here high-speed Digital Holographic Microscopy (DHM) records sperm flagellar waveforms and swimming paths in 4 dimensions (X, Z, and t). We find flagellar excursions into the Z-plane nearly as large as the envelope of the flagellar waveform projected onto the XY-plane. These Z-plane excursions travel as waves down the flagellum each beat cycle. DHM also tracks the heads of free-swimming sperm and the dynamics and chirality of rolling of sperm around their long axis. We find that mouse sperm roll CW at the maximum positive Z-plane excursion of the head, then roll CCW at the subsequent maximum negative Z-plane excursion. This alternating chirality of rolling indicates sperm have a chiral memory. Procrustes alignments of path trajectories for sequences of roll-counterroll cycles show that path chirality is always CW for the cells analyzed in this study. Human and bull sperm lack distinguishable left and right surfaces, but DHM still indicates coordination of Z-plane excursions and rolling events. We propose that sperm have a chiral memory that resides in a hypothetical elastic linkage within the flagellar machinery, which stores some of the torque required for a CW or CCW roll to reuse in the following counter-roll. Separate mechanisms control path chirality.

Introduction

Fertilization, the fusion of sperm and egg, is both the beginning of new life and the culmination of a complex selection of the single fertilizing sperm from among the millions delivered in the mammalian ejaculate. That selection imposes challenging barriers in the female reproductive tract opposed by swimming behavior of sperm that allows a small number to reach the site of fertilization [1–3] and for reviews see [4, 5]. Recognition that unaided fertilization requires sperm motility has prompted much study of sperm swimming behavior [3, 6, 7], of the flagellar waveforms that drive it [8–12], of the signaling pathways that control or modify it [13–21], and of the architecture of the flagellum [22–25], which imposes boundaries on all of the above. Here our attention is on the flagellar waveform and sperm swimming behavior.

Recent advances in holographic imaging have allowed quantitative examination of the 3D character of the swimming paths of mammalian [26–28] and invertebrate [29] sperm.

However, until now no studies report holographic imaging of the sperm flagellum and its dynamics.

We now apply high-speed holographic imaging to simultaneously examine flagellar waveform and swimming paths of sperm from the mouse, bull and human. Several features of the results are shared by sperm from these sources, but reconstructed image sequences of mouse sperm are particularly informative because they allow direct observation of the chirality of the rolling of sperm around their long axis.

Results

DHM records flagellar waveforms of free-swimming and of tethered mouse sperm

Sharply-focused XY-plane projections can be reconstructed easily from a time series of stored holographic images collected by DHM [30]. Fig 1A (left) shows the track of the centroid of the head of a typical free-swimming mouse sperm superimposed on the final frame of the video record of its time-series of reconstructed images (initial segment in S1 Movie). Such sequences of images also allow tracing of most of the flagellum. Fig 1A (right) shows traces from the first flagellar beat cycle (~0.3 s, indicating a beat frequency of ~3 Hz) and shows that the flagellar beat envelope was ~17 μm for most of its length.

Importantly, the paired x,y coordinates from these flagellar traces allowed interrogation of the holographic records for the associated Z-plane information. Fig 1B (for a better understanding please see also S2 Movie) shows that the Z-plane excursions traveled as a wave down the flagellum. The envelope of the traveling Z-plane wave (~14 μm) was nearly as large as that of the ~17 μm envelope of excursions into the XY-plane. The time course of travel of the peak Z-plane excursion down the flagellum (Fig 1C) indicated that the wave traveled at a mean linear rate of 421 ± 69 μm s^{-1} or 140 ± 23 μm per beat cycle. The mean rate for 6 cells from 3 experiments was 126 ± 16 μm per beat cycle, sufficient for the Z-plane wave to pass down the 125 μm length of the flagellum [31] once with each flagellar beat. A new, secondary Z-plane wave was initiated approximately midway through this sequence, presumably at the transition between the principal and reverse bends of the flagellum [32] observed in XY-plane projections.

The flagellar traces from 0.8 s of the video of the reconstructed record sampled in Fig 1A (S1 Movie) produced a matrix of x, y, z, and t values that allowed visualization of the flagellum in 4 dimensions. Fig 1D shows the flagellum at the time of the initial trace (at t = 0 s) and its projections onto the XY-, XZ-, and YZ-planes. The S3 Movie animates the sequence, providing a four-dimensional (4D) visualization of the flagellum of this free-swimming sperm. The traveling wave of Z-plane excursions (Fig 1B) is readily apparent in this video.

Sperm that are loosely attached (tethered) to the chamber surface at the head pivot about the point of attachment as the flagellum beats [14, 32]. The lower panel of Fig 1E shows that the path of the centroid of the head of a tethered sperm formed a small arc, but that the cell did not progress. As for free-swimming sperm, flagellar traces (upper panel in Fig 1E) provided x,y coordinates which yielded the associated Z-plane data from the holographic records. Fig 1F shows that, as for free-swimming sperm, the Z-plane excursions traveled as a wave down the flagellum. Also like free-swimming sperm, the envelope of the traveling Z-plane wave (~14 μm) was similar in size to the flagellar beat envelope (shown in Fig 1E). Unlike free-swimming sperm, the regression lines of the Z-plane excursions for the tethered cell possess only negative slopes from their origin at the proximal flagellum. Presumably this is a consequence of the cell being attached to the upper surface of the observation chamber. That

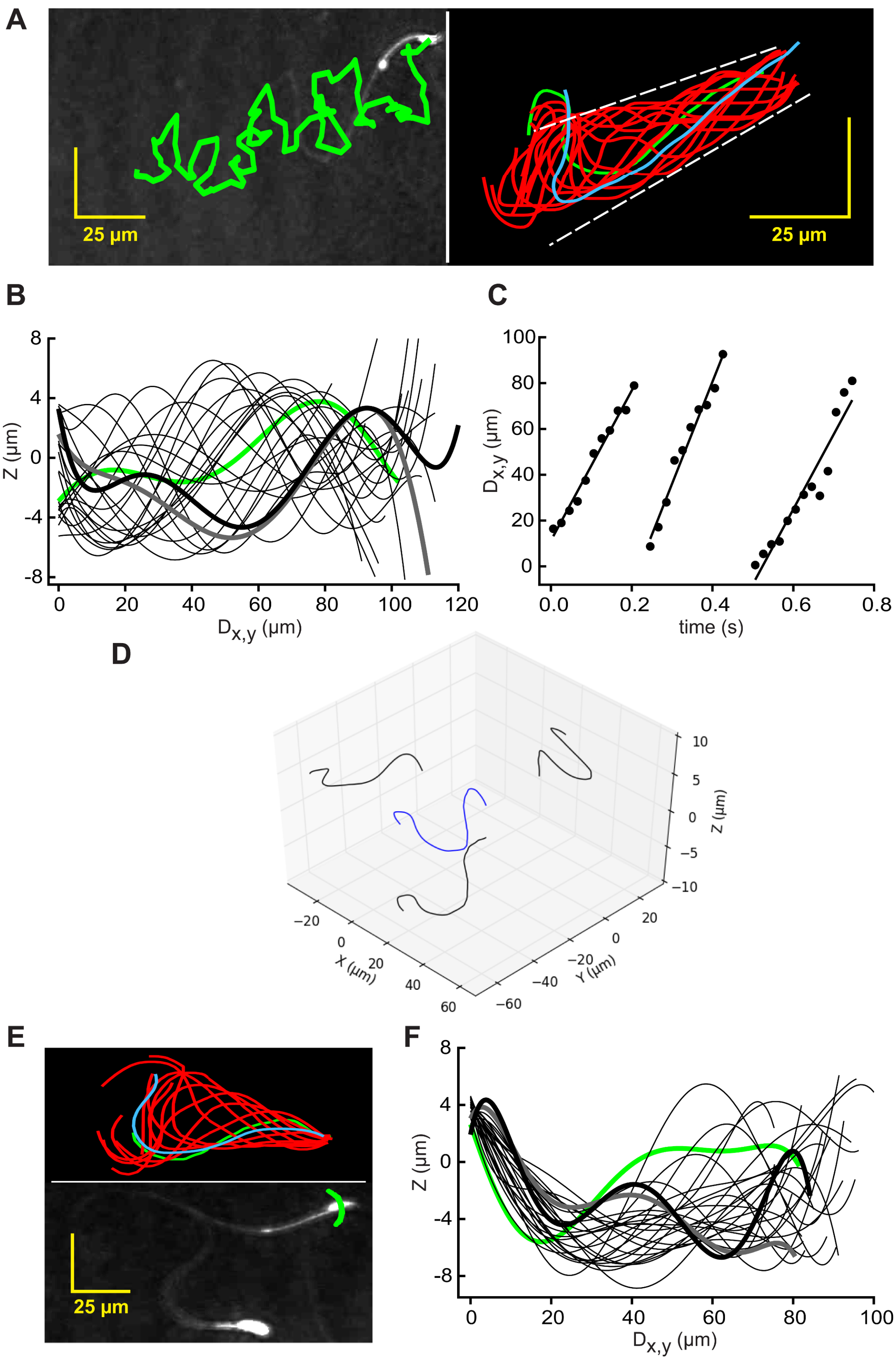
A
25 µm
25 µm
B
Z (µm)
8
4
0
-4
-8
0
20
40
60
80
100
120
$D_{x,y}$ (µm)
C
$D_{x,y}$ (µm)
100
80
60
40
20
0
0.0
0.2
0.4
0.6
0.8
time (s)
D
10
5
0
−5
−10
Z (µm)
−20
0
20
40
60
X (µm)
20
0
−20
−40
−60
Y (µm)
E
25 µm
F
Z (µm)
8
4
0
-4
-8
0
20
40
60
80
100
$D_{x,y}$ (µm)

Fig 1. Traced XY-Plane projections reveal 4D character of flagellar waveforms of free-swimming and tethered mouse sperm. (A) (left) Path of a free-swimming sperm (green) overlaid on the reconstructed XY projection of the final frame of the 2.4s holographic record of S1 Movie; (right) Flagellar traces sampled at 50 Fps for the first beat cycle (~0.29 s). First trace (green), final trace (blue). Waveform envelope (dashed line) is ~17 μm wide. (B) Excursions into the Z-plane reported by interrogation of the holographic records with the x,y coordinates from the traces in the right panel of A. The Z-plane data (sampled at 50 Fps) was smoothed by fitting to 7th order polynomials, then plotted against the calculated distance along the XY projection of the flagellum (Dx,y). The polynomial regression was used to generate a best fit curve from nonlinear data. Polynomial fits are widely accepted to describe the shape of a flagellar waveform [33–35]. It helps to visualize the data, to interpolate gaps between data points and to remove noise from the determination of z values (see also S1 Fig).

https://doi.org/10.1371/journal.pone.0199678.g001

conjecture was supported by the finding that no other objects in the same field of view had Z-plane coordinates greater than that of the centroid of the head of this cell.

We conclude that the flagellar waveform of free-swimming mouse sperm is not planar but instead has a substantial Z-plane component produced by waves of Z-plane excursions that accompany both the principal and reverse bends observed in XY-plane projections. Because Z-plane waves also are observed for tethered sperm, rolling of sperm around their long axis is not required for their production.

Fitted data from frame 0 is green, that of frames 3, 6,. . .51 are light black, and those of the last 2 frames (54 and 57) are heavy gray and black. (C) Time course of the travel of peak Z-plane excursions down the flagellum. Least-square regression lines report rates of travel. (D) The flagellum of the same cell (blue) and its projections (black) onto XY-, XZ-, and YZ-planes at t = 0 s (the first frame of the 4D animation of S3 Movie). (E) (lower panel) Path of the head of a tethered sperm (green) overlaid on the XY projection of the final frame of a 3s record sampled at 100 Fps; (upper panel) flagellar traces sampled at 100 Fps for the first beat cycle (~0.16 s). First trace is green, final trace blue. (F) Smoothed excursions into the Z-plane reported by interrogation of the holographic records with x,y coordinates from the traces in the upper panel of E. Fitted data from frame 1 is green, of frames 3, 5,. . .21 are light black, and those of last 2 frames (23 and 25) are heavy gray and black.

DHM tracks swimming paths and rolling behavior of mouse sperm

Extraction of Z-plane information for the flagellum (Fig 1) required manual tracing to obtain x,y coordinates for interrogation of the holographic records. The process was simpler for the head of the sperm; its larger size and more refractile properties provide a stronger signal which allows the DHM Koala software to directly track the head in the X-, Y-, and Z-planes. The tracking is performed frame by frame on numerically calculated projection images of the focal planes within the reconstruction distance. For a different free-swimming cell (secondary segment of S1 Movie) Fig 2A–2C show that 14 μm-long axes allowed visualization of all points along the 2.2s swimming path. Fig 2D is a 3D representation of this path, rotated to view the approaching sperm head-on. The initial portion of the accompanying S4 Movie animates this rotation and the path progression. Color coding of the Z-plane excursions emphasizes the complex, repetitive character of the swimming path.

The head of mouse sperm has assigned dorsal and ventral surfaces (with a dorsal acrosome and ventral spur). Therefore the head has assigned left and right "cheeks" and the orientation of the cell can be described as either "right-cheek downmost" (RCh) or "left-cheek downmost" (LCh). Examination of the time series of reconstructed XY-plane images of the cell of Fig 2A–2D showed that rolling of the sperm from RCh-downmost to LCh-downmost (and *vice versa*) produced transient increases in intensity of light reflected from the sperm head (upper panel in Fig 2E), consistent with prior work using conventional bright-field microscopy [6]. In addition, the higher magnification and shorter illumination times used here for DHM allowed visualization of the chirality of rolling indicated by the curved arrows. Consistently, sperm

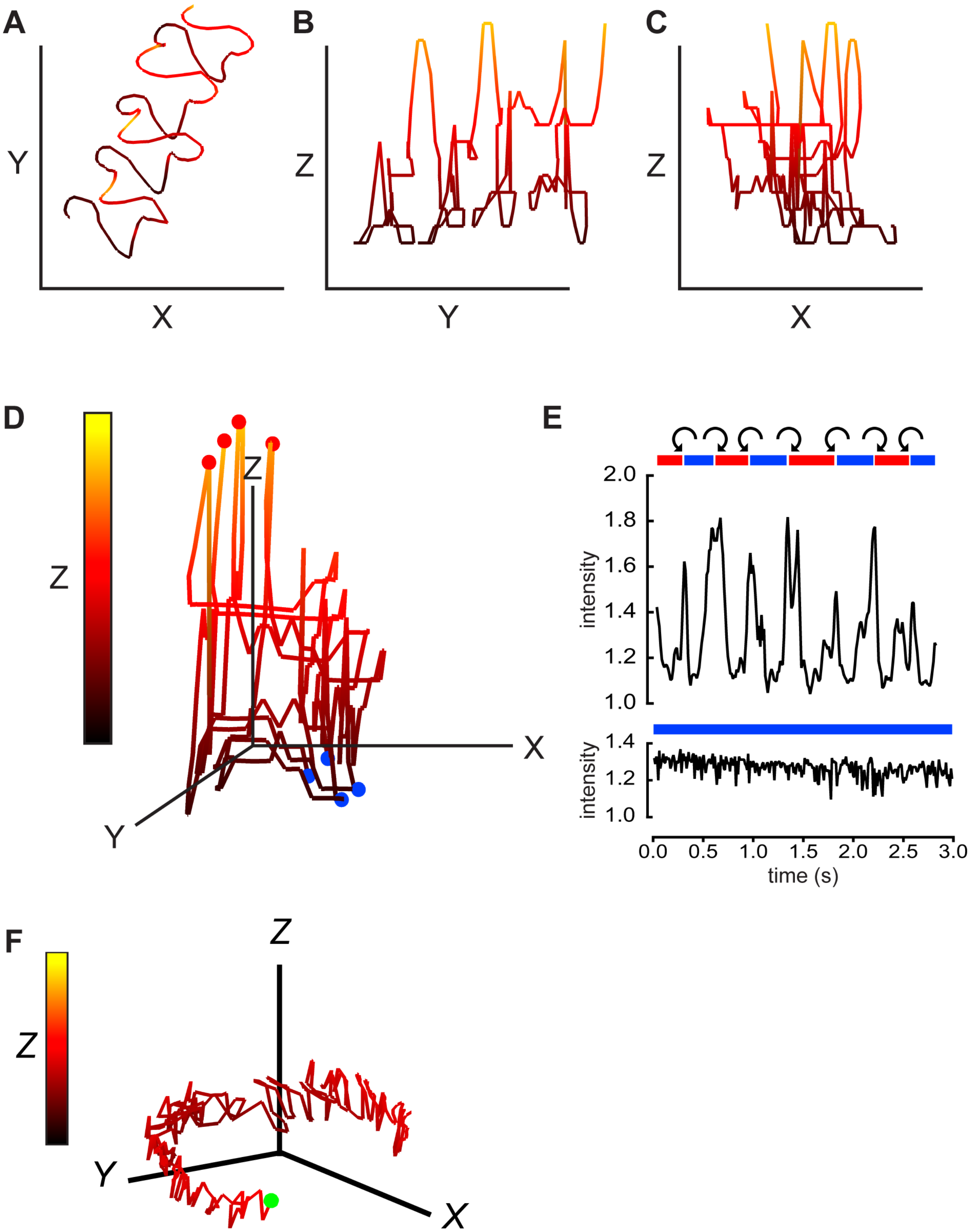

Fig 2. Digital holographic imaging also provides 4D description of sperm swimming trajectories and rolling behavior. The path of the head of another free-swimming mouse sperm with a linear averaged trajectory was projected onto the XY-plane (A), the YZ-plane (B), and the XZ-plane (C). Equal scaling (14 μm) of all axes. Z-plane excursions are also color coded as indicated by the vertical bar in panel D below. The 2nd segment of S1 Movie is the XY projections of the 2.2s holographic record. (D) A 3D view of the swimming path, red balls indicate path locations where the cell rolls from "LCh" to "RCh" orientation, blue balls indicate locations where cell rolls "RCh" to "LCh". (E) (upper panel)

Red and blue bars indicate periods of "RCh" and "LCh" orientation for this cell. The orientation is indeterminate in the short white areas. Curved arrows indicate CW or CCW rolling around the long axis of the cell. Also shown is the oscillating intensity of light scattered from the head of the rolling sperm (relative to background from a cell-free area). In the lower panel, a different, non-rolling sperm maintains a LCh orientation and lacks periodic fluctuations in intensity of reflected light. (F) A 3D view of the circular path of this non-rolling cell. Green ball marks the starting point. Scale bars 14 μm. Z-plane excursions color coded as in D.

https://doi.org/10.1371/journal.pone.0199678.g002

rolled CCW at the RCh- to LCh-downmost transition, then always followed with a CW roll at the transition from LCh- to RCh-downmost. In independent, blind trials a panel of 3 observers assigned the same CW or CCW chiralities to the first 2 rolls occurring for sperm in each of the same 10 representative video reconstructions of free-swimming sperm. Each observer also examined rolling over the entire length (1–3 s in duration) of 1 (randomly assigned) video from the group of 10. All observers reported that CW rolls invariably followed CCW rolls (and *vice versa*). We conclude that sperm have a chiral memory.

The sperm examined in the lower panel of Fig 2E remained in the LCh-downmost orientation for the entire 2.8s recording. As expected from past work [6] it did not show the transient increases in intensity of reflected light that report rolling. Also as expected from its LCh orientation, its swimming path circled CW (Fig 2F). The Z-plane excursions for this non-rolling cell were much smaller than for the rolling cell of Fig 2A–2D. Nevertheless, cells tethered at the head to prevent rolling still show large Z-plane excursions of the flagellum (Fig 1F). This finding is consistent with the observations of movement at low Reynolds numbers where inertial forces can be neglected. As a consequence the source of all movement including the rotation of the head is flagellar beating [3, 36].

Comparison of the timing of the transitions between RCh and LCh configurations in Fig 2E with that of the 3D path profile of Fig 2D revealed that rolling from RCh- to LCh-downmost occurred at the peak positive excursion into the Z-plane and that LCh- to RCh-downmost rolling occurred at the peak negative excursion into the Z-plane. The timing of these rolling events are marked by the red and blue circles in Fig 2D. The period between 2 circles of the same color constitutes a "roll cycle".

The correlations between rolling events and Z-plane excursions make it somewhat easier to see the irregularly helical nature of the swimming path. The accompanying S4 Movie animates a rotation of Fig 2D that allows a clearer viewing of its 3D character. A rolling ball then reiterates the time course of this sequence, showing the CW chirality of the swimming path as viewed facing the oncoming sperm. Another supporting Movie (S5 Movie), showing an animation of a free swimming sperm with a clockwise path chirality, provides additional clarification of a helical trajectory structure.

Chirality of mouse sperm swimming paths from Procrustes alignments

Although rotation of the matrix of x, y, z coordinates allowed visualization of the chirality of the swimming path for the cell of Fig 2A–2D, the process was cumbersome and required subjective judgments. Seeking a more automated, objective alternative we turned to Procrustes alignment, a favored tool for morphometric analysis [37]. Procrustes analysis, named after a villainous bandit from greek mythology ("the stretcher"), is a statistical method that is used primarily for shape analysis in biological or medical data. The aim is a matching between two or more shapes via superimposition while a shape is defined as a configuration of data points (landmarks) organized in a matrix. The analysis is implemented by symmetry operations with the purpose to minimize the sum of distances (Procrustes distance) between corresponding data points from each shape. Mathematically, a translation is followed by a rotation and an isotropic rescaling [38]. Fig 3A shows the XY-plane trajectory of the cell of Fig 2A–2D rescaled to

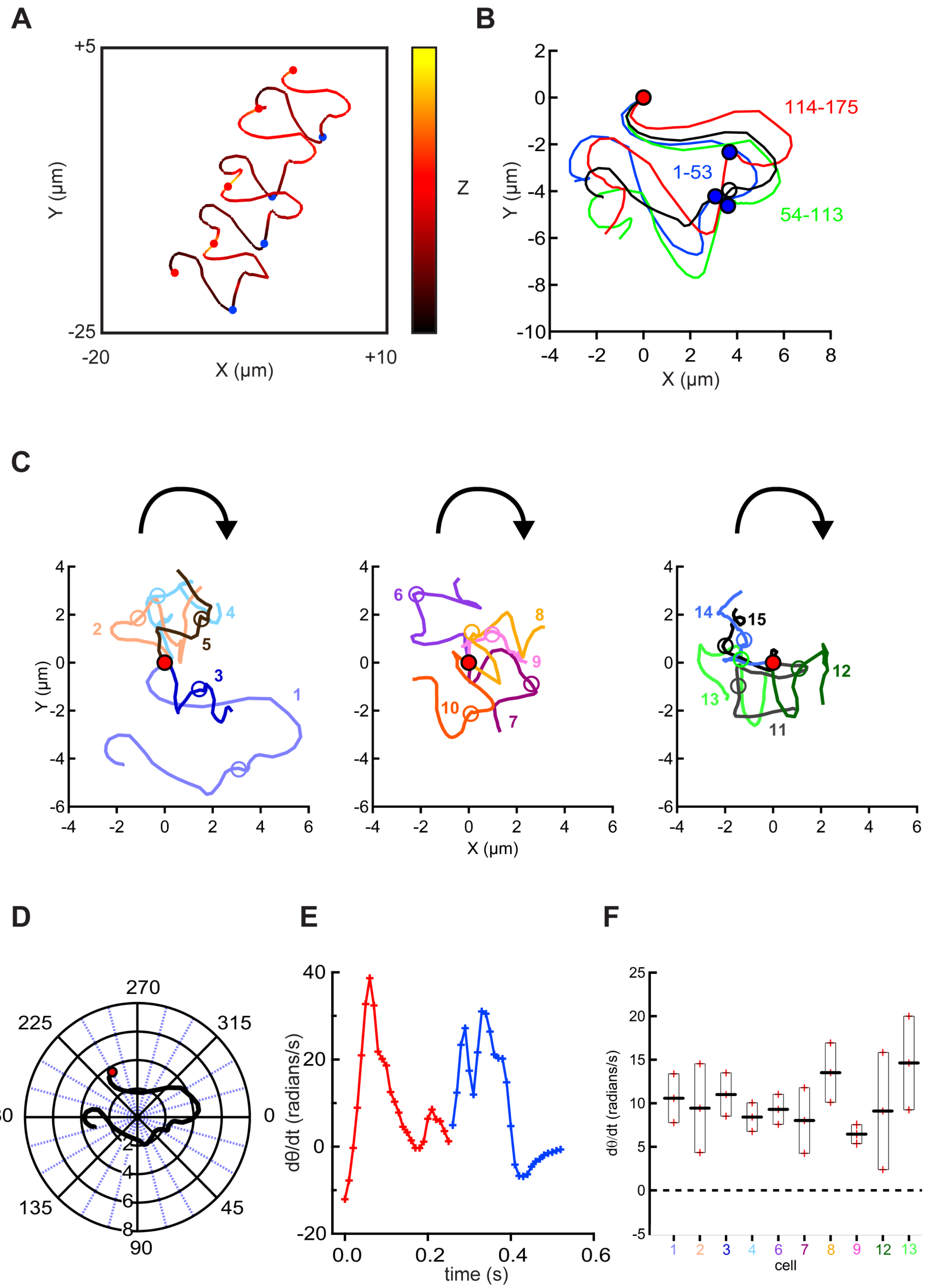
A
+5
-25
-20
+10
X (µm)
Y (µm)
Z
B
114-175
1-53
54-113
X (µm)
Y (µm)
C
X (µm)
Y (µm)
D
270
225
315
180
0
135
45
90
E
dθ/dt (radians/s)
time (s)
F
dθ/dt (radians/s)
cell

Fig 3. Procrustes alignment of head trajectories reveals conserved chirality of swimming paths. (A) A 3D view of the trajectory of the head of the free-swimming sperm of Fig 2D. Balls indicate rolling of sperm from LCh to RCh (red) and RCh to LCh (blue). (B) Procrustes alignment of the XY-plane trajectories for the 3 roll cycles (starting immediately after a RCh to LCh roll, proceeding through a RCh to LCh roll, and continuing until a new roll then back to RCh) spanning the indicated range of frames for this cell. Black trace is their average. (C) Averaged Procrustes alignments for cells that rolled CW then CCW during 2–3 roll cycles. Each averaged alignment was translocated to place the initial point at the origin (5 cells in each of the 3 panels). The curved arrow indicates path chirality judged by visual assessment. (D) The averaged alignment of cell 1 from panel C (also examined in panels A and B, and in Fig 2) was translocated to place its origin at the mean X and mean Y values, then transformed into polar coordinates. (E) Time course of the time derivative of the angular component of the transformed alignment (dθ/dt) for cell 1. Red and blue lines and crosses indicate segments of RCh and LCh orientation. (F) Determined dθ/dt (mean ± s.e.m.) for 10 cells from panel C (same color coding).

https://doi.org/10.1371/journal.pone.0199678.g003

emphasize that it comprises 4 cycles of rolling from RCh- to LCh-downmost, then back again (the segments between the red circles). To apply Procrustes alignment (Fig 3B), the beginning x,y coordinates of each of the indicated cycle segments were translocated to the origin. Then scaling and rotation were adjusted to maximize similarity of each segment to that of the first cycle. Finally the adjusted traces were averaged. The similarity of the 3 aligned cycles strongly indicates that their complex trajectories did not result from random noise. We also note that each aligned cycle begins with a predominantly CW curvature of the XY-plane path, then showed a sharp, transient, midpoint transition when the cell rolls from the RCh- to LCh-downmost configuration.

The averaged aligned trajectories of the 15 cells shown in the 3 panels of Fig 3C differ in detail, but all show an initial CW curvature with a midpoint transition when the cell rolls to the LCh configuration. Because the swimming path initially moved downward in the Z-plane while the cells were in the RCh configuration then moved upward after rolling to the LCh, the trajectories appear irregularly helical with the CW chirality shown in the animated S4 Movie.

Seeking an objective measure of path chirality, we translated the averaged Procrustes alignment for cell 1 of Fig 3C into polar coordinates (Fig 3D) then examined the time course of changes to its angular component (θ). The time derivative of theta (dθ/dt) then reported how rapidly the curvature of the path changed and its CW or CCW direction. Fig 3E shows that for this cell dθ/dt was the largest at the beginning and near the midpoint of the trajectory when the cell rolls from LCh to RCh and then from RCh to LCh. More importantly, the data of Fig 3F show that the mean dθ/dt for the time course of the averaged Procrustes alignment of cell 1 had a positive value (10.6 ± 2.8 radians/s) indicating a CW chirality. The magnitude of mean dθ/dt range ranged from 6.5 to 14.6 radians/s for the 10 cells examined in Fig 3F, similar to the ~2-fold range of their estimated flagellar beat frequencies. More importantly, all had positive values providing quantitative verification that a CW path chirality is a shared property of mouse sperm examined under the conditions described here.

Flagellar waveforms and path trajectories of free-swimming bull and human sperm

In videos of a representative free-swimming bull sperm reconstructed from DHM records, the sperm head showed a linear averaged trajectory with staggering lateral excursions and alternating periods of forward and retrograde progression (left panel in Fig 4A, initial segment of S6 Movie). Averaged path speeds of 149 ± 12 μm s^{-1} and progression of 57 ± 12 μm s^{-1} (n = 27 cells, N = 3) resembled those of mouse sperm examined under similar conditions (path speed of 168 ± 25 μm s^{-1} and progression of 43 ± 11 μm s^{-1}, n = 20 cells, N = 5). Comparison of the left panels of Figs 4A and 1A indicated that trajectories for bull sperm had smaller lateral excursions than those for mouse sperm, presumably a consequence of their shorter flagella and more symmetrical flagellar beat. Comparison of the right panels of Figs 4A and 1A shows that for bull and mouse sperm the envelopes of the flagellar traces had a similar size (~15 μm).

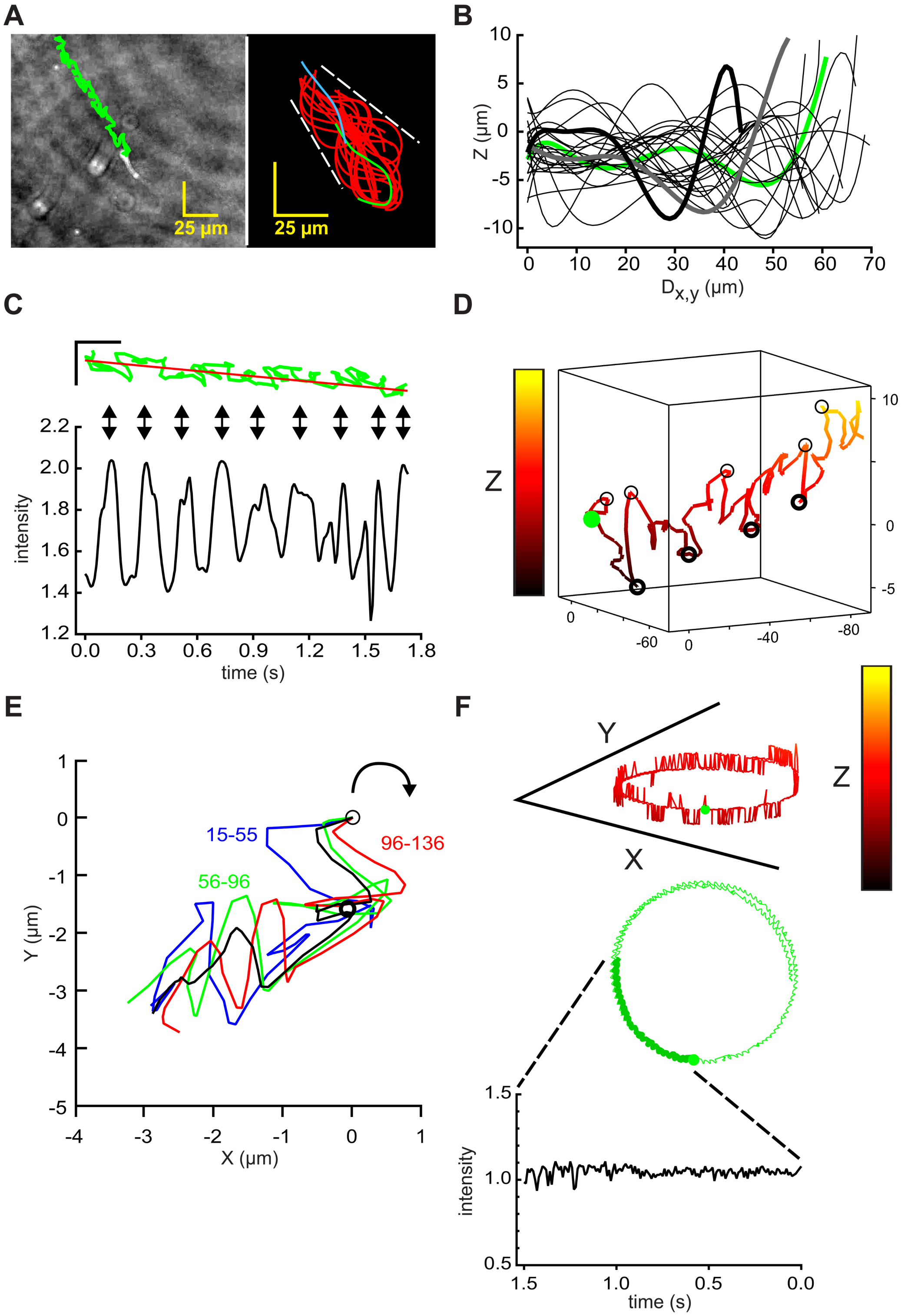

Fig 4. Holographic imaging provides 4D descriptions of flagellar waveform and swimming trajectories of bull sperm. (A) (left) Path of the head of a free-swimming bull sperm (green) overlaid on the XY projection of the final frame of a 1.8s holographic record sampled at 100 Fps, scale bars 25 μm; (right) flagellar traces sampled at 50 Fps for the first 2 beat cycles (~0.5 s). First trace (green), final trace (blue). Waveform envelope ~25 μm. (B) Z-plane excursions, by interrogation of the holographic records with x,y coordinates from the traces in right panel of A. Z-plane data sampled from alternate frames, smoothed by fitting to 7th order polynomials, plotted against calculated distance along the XY projection of the flagellum

(Dx,y). Data from frame 0 is green, from frames 2, 4, . . .46 are black, from frames 48 and 50 heavy gray and black. (C) Rotated version of the time course of swimming path in green with its regression line in red, scale bars 25 μm (upper panel). Vertical arrows show alignment with peaks in the intensity of light scattered from the head (relative to cell-free background, lower panel) intensity. (D) 3D view of swimming path of sperm head, green circle marks beginning, light and heavy circles mark locally-maximal positive and negative Z-plane excursions. X,Y, and Z scales 140, 140, and 14 μm. (E) Procrustes alignment of indicated path segments, black is averaged alignment. Light circle marks origin, heavy circle marks midpoint of the averaged alignment. (F) 3D view of the circular path of another cell projected onto the XY-plane (upper panel), green circle marks beginning. The middle panel shows XY-plane projection of the swimming path (X and Y scales 140 μm, Z scale 14 μm). Lower panel, time course of optical signal from the head during initial 1.5 s path segment (heavy green in middle panel).

https://doi.org/10.1371/journal.pone.0199678.g004

The matrix of x,y coordinates for the flagellar traces of Fig 4A allowed extraction of the corresponding Z-plane coordinates. Fig 4B shows that, as for mouse sperm, the size of bull sperm flagellar Z-plane excursions (~15 μm) was similar to that of the flagellar envelope in the XY-plane, and that Z-plane excursions traveled as waves down the flagellum.

Also like mouse sperm, bull sperm have a highly refractile head which produces periodic increases in intensity of reflected light when the cell rolls (Fig 4C and initial segment of S6 Movie). Unlike mouse sperm, bull sperm (and sperm of most other eutherian mammals) do not have distinguishable dorsal and ventral surfaces under the DHM setup, so it was not possible to assign chirality to the rolls that could be seen by visual examination and whose dynamics were reported by increases in the intensity of the optical signal.

Fig 4D is a 3D representation of the path of the head of the sperm of Fig 4A–4C, shown with equal X- and Y-axes. An expanded Z-axis and rotation of the viewing angle were chosen to show that the timing of the peak intensity signals (Fig 4C) correlated alternately with locally maximal positive Z-plane excursions (light circles) and locally maximal negative Z-plane excursions (heavy circles). If bull sperm also possess the chiral memory found for mouse sperm in Fig 2, then these open and closed circles would also mark alternating CW and CCW rolling and counter-rolling events.

Fig 4E applied Procrustes alignments to the indicated segments of the XY-plane path trajectory that spanned 2 intensity peaks, signaling 2 rolling events, and which together formed a roll cycle. The average of the alignments indicated that the XY-plane trajectory curved CW for those segments which occurred after a maximal positive Z-plane excursion. Such CW curvature indicates that the path of this bull sperm had a CW chirality, like that of mouse sperm (Fig 3C and 3D).

In contrast to the linear averaged trajectory of the cell of Fig 4A–4E, the cell of Fig 4F completed a circular (~120 μm diameter) trajectory during ~7s of a 10s recording (second segment of S6 Movie). In the initial portion of this record (heavy green line, middle panel of Fig 4F) the path speed was 122 ± 4 μm s^{-1}. The intensity of reflected light (lower panel) showed no periodic oscillations, consistent with an absence of rolling. We conclude that, as for mouse sperm, an absence of rolling allowed the slightly asymmetrical flagellar beat of bull sperm to produce a circular trajectory.

The 3D representation of the path of the sperm head (upper panel of Fig 4F) shows that Z-plane excursions for this circling cell were much smaller in size (nearly all are steps of 1.4 μm above or below the mean Z axis location) than for the cell with a linear trajectory (Fig 4A–4D). Because many of the indicated excursions spanned only a single time point, it is likely that they resulted from random noise. The technique of digital holographic microscopy features a numerical processing of holograms for the reconstruction of quantitative phase images. This calculation allows to compensate experimental noise such as time drift or vibration [39]. In addition to that, there are no lens aberrations apparent. A possible source of noise can be a weak contrast of the specimen, for example of the tip of the flagellum, which affects directly

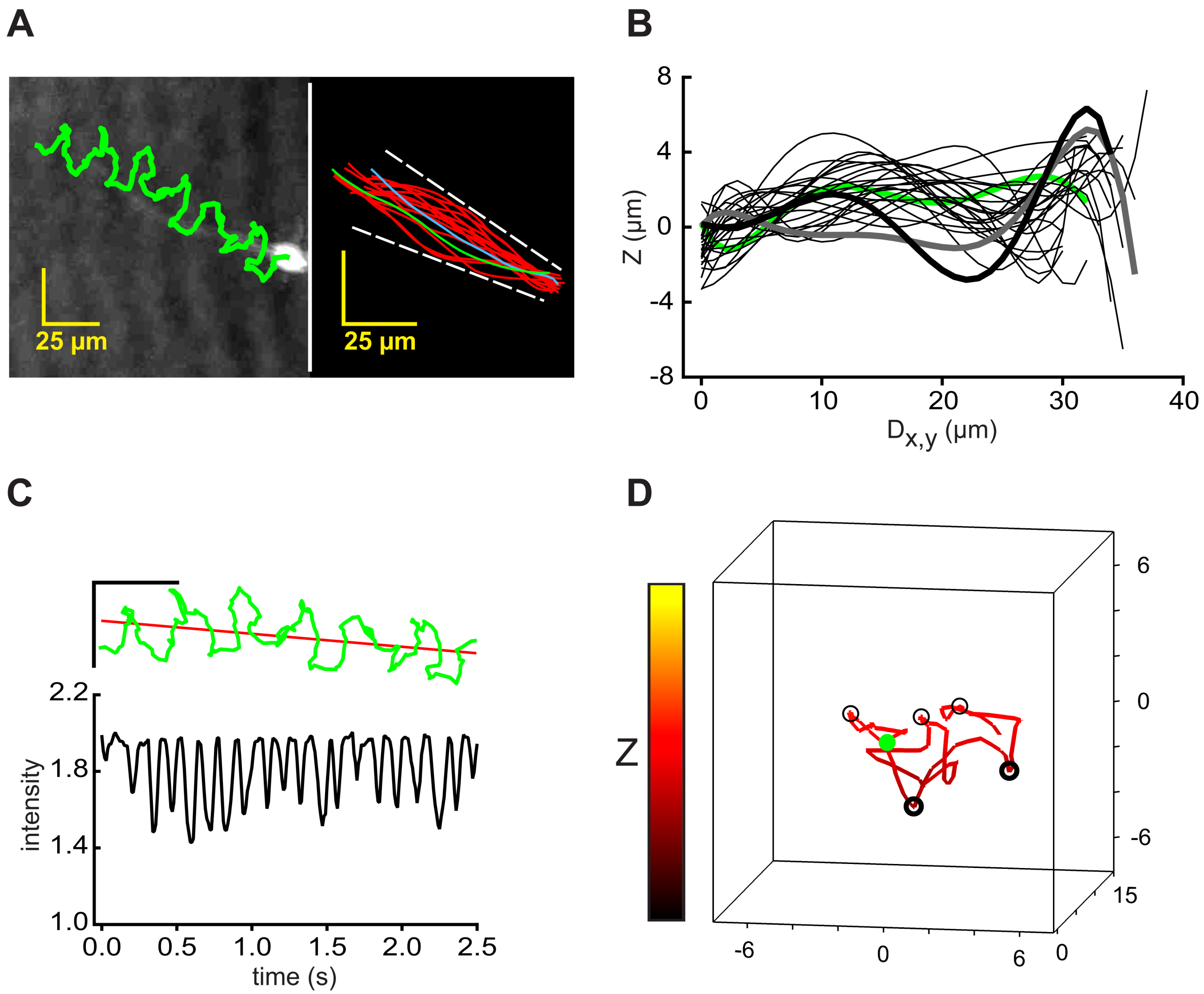

Fig 5. Holographic imaging provides 4D description of flagellar waveform and swimming trajectories of human sperm. (A) (left panel) Path of the head of a free-swimming human sperm (green) overlaid on the XY projection of the final frame of a 2.5s holographic record sampled at 100 Fps; (right panel) Flagellar traces sampled at 50 Fps for the first beat cycles (~0.29 s). First trace (green), final trace (blue). Waveform envelope is ~15 μm. (B) Excursions into the Z-plane reported by interrogation of the holographic records with the x,y coordinates from the traces in the right panel of A. The Z-plane data sampled at 50 Fps was smoothed by fitting to 7th order polynomials, then plotted against the calculated distance along the XY projection of the flagellum (Dx,y). Fitted data from frame 1 is green, that of frames 3, 5, . . .55 are black, and that of frames 57 and 59 are heavy gray and black. (C) (Upper panel) A rotated version of the time course of swimming path in green with its regression line of the trajectory in red, scale bars are 25 μm. (Lower panel) Time course of relative intensity of light scattered from the head. (D) A 3D view of the swimming path of the sperm head, green circle marks beginning, light and heavy circles mark locally maximal positive and negative Z-plane excursions.

https://doi.org/10.1371/journal.pone.0199678.g005

the signal to noise ratio. The limited frame rate of the camera in correlation with the velocity of the sperm can also cause an aberration. Finally, the reconstruction itself defines the number of possible z values and therefore the z resolution.

Fig 5A (left) shows the track of the centroid of the head of a typical human sperm superimposed on the final frame of the video record of its time-series of reconstructed images (third segment in S6 Movie). Tracking of this cell indicated a path speed of 47 ± 2 μm s^{-1} (overall average = 64 ± 11 μm s^{-1}, n = 20 cells) and progression of 13 μm s^{-1} (overall average = 20 ± 6 μm s^{-1}). Fig 5A (right) shows traces from the first flagellar beat cycle (~0.14 s, corresponding

to a beat frequency of ~7 Hz) which indicated that the flagellar beat envelope was ~10 μm for most of its length. The flagellar Z-plane locations, obtained using the x,y coordinate matrix from a longer series of flagellar traces, showed traveling waves of Z-plane excursions (Fig 5B) whose size was also ~10 μm.

Human sperm have heads that are quite variable in morphology [40] and that have more refractile surfaces than those of mouse and bull sperm. Those properties create more variability in the optical signals produced by rolling. The dynamic range of these signals (lower panel in Fig 5C) was also smaller than the optical signals from mouse and bull (Figs 2E and 4C) and their alignment with changes in path trajectory (upper panel in Fig 5C) was more difficult to see.

Fig 5D is a 3D representation of the initial 1 s of the path of the head of the sperm of Fig 5A–5C, shown with equal X,Y, and Z axes. Rotation of the viewing angle was chosen to show the timing of the maximal positive Z-plane excursions (light circles) and maximal negative Z-plane excursions (heavy circles). Without a more direct indication of rolling we have not attempted to assign chirality to the swimming path. No circular paths were observed for any of the 20 cells tracked in this study. Therefore, we could not show that an absence of rolling correlated with circular swimming paths for human sperm.

In summary, analysis of the swimming behavior of mouse sperm is most informative. Analysis of bull sperm swimming behavior indicates strong similarities to that of mouse sperm. With currently available methodology, analysis of human sperm swimming behavior is more difficult and less informative, but represents a starting point for future exploration.

Discussion

Currently we have quite detailed knowledge of the architecture of the mammalian sperm flagellum and of the 9+2 axoneme that drives it [23, 25, 41, 42]. However, we know much less about how this machinery initiates and propagates the flagellar wave [8, 9, 11, 12, 43–45]. In fact, until now we have lacked a detailed picture of the flagellar waveform itself. The present work applies high-speed DHM to the beating flagellum of mouse, bull, and human sperm and provides quantitative descriptions of the 3D flagellar waveforms and their dynamics.

Although early work recognized that sperm flagellar waveforms are not entirely planar [46, 47], the size of excursions out of the XY-plane was only estimated from the loss of focus observed with high numerical aperture objectives [48–50] or from the width of the intensity maxima along line scans across the flagellum [34]. With DHM we now see that the flagella of free-swimming sperm make waves of excursions into the Z-plane that have approximately the same amplitude as the waves in the XY-plane. We also see that initiation of flagellar Z-plane waves coincides with initiation of the principal and reverse flagellar bends in the XY-plane, and that the Z-plane waves travel at constant speed down the flagellum. These new, quantitative findings will place supplemental constraints on proposals to explain the basis of flagellar bend formation.

We also find that Z-plane waves are still produced by mouse sperm which are prevented from rolling by tethering of their heads to the chamber surface. Therefore, production of Z-plane waves is not constrained by rolling of sperm around its long axis. If the torsion required for rolling results from the travel of the Z-plane waves down the flagellum, then that torque should be present and measurable at the head of the tethered sperm. Allen [51] has described atomic force microscopy techniques that may be applicable to such measurements.

We show that for those free-swimming mouse sperm which do roll, each roll produces a transient increase in intensity of the light reflected from the sperm head. That optical signal serves as a useful marker of the timing of rolling events observed here in reconstructed

projection images and in previous work [3, 6] using conventional microscopic imaging. Monitoring the chirality of the rolling events by eye, we find that CW and CCW rolling alternate to form a roll-counter-roll cycle. The higher numerical aperture objectives used here provide more certainty to the assignments of the chirality of rolling than to the assignments made in that previous work. However, a less subjective, quantitative method still remains desirable.

Ishijima and coworkers studied the special case of sperm swimming vertically upwards against a coverslip. In one report [48] 76% of the heads of hamster sperm rolled CCW. Similar proportions of bull and human sperm also rolled CCW. However, another study [52] found that most hamster sperm rolled CW as seen from the anterior. For mouse sperm also swimming upward against a coverslip, Woolley [53] reported that all rolled CW. That finding contrasts with our observations that for free-swimming mouse sperm each CW roll is followed by a CCW roll and *vice versa*. An explanation for these different findings is not apparent. Perhaps head-on encounters with the coverslip selectively suppress CW or CCW rolling.

To explain the consistently alternating pattern of CW and CCW rolls observed for mouse sperm, we propose that the sperm have a chiral memory which stores a portion of the torque that is required for rolling for reuse in the following counter-roll. Early work with sea urchin sperm also indicated the presence of a chiral memory. Specifically, Gibbons and coworkers [54] saw that when the sperm head was held in the tip of a vibrating micropipette, the natural plane of the flagellar beat changed to that of the plane of imposed vibration. When the vibration ceased after several complete revolutions of the vibration plane, the flagellar beat plane then spontaneously "unwound" back to the initial, natural plane of the beat [55, 56]. Those observations suggested the presence of an elastic restraining force. If such elastic forces in sea urchin and mouse sperm have a common basis, then the responsible component(s) must not reside in the outer fibers or fibrous sheath which are absent from echinoderm sperm.

Although the present study is the first to apply digital holographic imaging to examine swimming paths of mouse and bull sperm, holographic imaging was used in previous high-throughput studies of the paths of human [28] and horse [26, 27] sperm. That past work reported that "planar" trajectories were mostly found for those sperm (~40% of the population) near the boundaries of the chamber. It seems likely that such trajectories are segments of the circular swimming paths that we find for non-rolling sperm. In previous findings we could show, that non rolling mouse sperm remain either on the left or the right cheek. Through the asymmetrical shape of the mouse sperm head those sperm become circular swimmers with either a CW (left cheek orientation) or CCW (right cheek orientation) [6]. Another ~25% of the population were described as having "twisted ribbon" trajectories which the authors recognized as helicoid surfaces. The irregular helical trajectories that we observe for the most free-swimming sperm probably also would fall on, or near, such helicoid surfaces. Modeling of helicoid trajectories remains to be explored.

Nosrati and coworkers [7, 57] found that some bull and human sperm produced near-field fluorescence signals whose origin was attributed to planar flagellar waves of cells swimming within 1 μm of the chamber surface. However, we find that the flagella of free-swimming bull sperm, like those of the mouse, produce traveling waves of excursions into the Z-plane. We show that these Z-plane excursions persist even when mouse sperm are tethered at the head to the chamber surface. Although tethered bull and human sperm were not observed with the protocols used here, it is likely that Z-plane excursions will also be shown to persist when protocols become available to tether bull and human sperm. If so, then either some free-swimming sperm of bull and human have the ability to suppress flagellar Z-plane excursions, or the near-field signals have been misinterpreted.

The swimming behavior of the green algae *Chlamydomonas* also has been much studied. It uses a breaststroke waveform of its two flagella to pull the cell body towards brighter

illumination. Recent work by Geyer and coworkers [43] showed that the breaststroke waveform has separable static and dynamic components. As viewed in XY-plane projections, the dynamic component is a traveling wave that provides propulsion and the static component is a circular arc that controls swimming direction. A flash of high intensity light evokes an avoidance response produced by suppression of the static component, which converts the breaststroke waveform to a symmetric sinusoidal waveform that pushes the cell away from the source of the noxious stimulus.

For sperm, the XY-plane projections of the flagellar waves are quite sinusoidal under the "resting conditions" used in the present study, indicating that the dynamic component of the waveform dominates. Sperm produce a much more asymmetric waveform after exposure to "capacitating conditions" [6, 14] which apparently evoke a Ca^{2+}-mediated engagement of the static component of the waveform. Permeabilized or excised flagella retain the ability of Ca^{2+} to modulate waveform asymmetry [58], indicating that Ca^{2+} acts on a component of axoneme itself. The molecular identity of the Ca^{2+}-sensitive component(s) is not known.

The static and dynamic components of the algal flagellar waveform can be separated genetically. The dynamic component exists in the absence of the static component for the *Chlamydomonas mbo2* mutant [43]. Similarly, sperm of CatSper-null mice have a sinusoidal waveform with low asymmetry, but do not develop the highly asymmetric hyperactivated waveform [14]. Hence the various CatSper-null mutants could be considered as "waveform phenocopies" of the *mbo2* mutant, even though the sperm and algae mutants almost certainly disrupt expression of unrelated target proteins.

It has not been shown that *Chlamydomonas* flagella make waves of Z-plane excursions like those that travel down the sperm flagellum, as shown here. The smaller size and faster beat frequency of the algae flagella (and of motile cilia found elsewhere) probably will prevent examination by holographic imaging using currently available instruments. Meanwhile, the issue of waveform propagation has been addressed by 2D modeling studies based on various predictions of how mechanical forces generated by axoneme act to regulate its motor proteins [59]. The results indicated that a curvature control model with axonemal twist produced the best fit to the waveforms observed for both the algae and for bull sperm. This led to the conclusion that the dynein motors drive both sliding of adjacent microtubule doublets and generate torsional forces that twist the axoneme, thereby creating a nonplanar component of the waveform. Our work here provides measurements of the magnitude and dynamics of the Z-plane excursions presumably produced by such twisting.

In summary, DHM has given us new quantitative insights into both the flagellar waveform and the swimming behavior of sperm. Application of DHM to mouse sperm has been particularly informative, opening new areas for exploration of how sperm generate, store, and reuse the torsional forces that underlie the flagellar waveform.

Materials and methods

Chemicals

All chemicals were purchased from Sigma-Aldrich.

Sperm preparation and media

After isoflurane anesthesia and cervical dislocation of NMRI mice (Charles River Labs, Wilmington DE, USA), cauda epididymides and vasa deferentia were excised and sperm released and processed as described previously [6]. Briefly, sperm were allowed to swim out into medium HS (in mM: 135 NaCl, 5 KCl, 2 $CaCl_2$, 1 $MgCl_2$, 20 Hepes, 5 glucose, 10 DL-lactic acid and 10 pyruvic acid, pH 7.4) for 20 min at 37˚C and 5% CO_2. After two washing steps

(sedimentation for 3 min at 400 x g) sperm were resuspended to a final concentration of 1–2 x 10^7 cells/ml in HS medium with or without 5 mg/ml BSA and stored at RT. Bovine ejaculated sperm, collected from a Holstein Friesian bull (HB-No 678525), were the generous gift of Rinder-Union West eG (Münster, Germany), and transported from a local branch facility to our lab within 2 h of collection. A panel of 3 donors provided ejaculated human sperm, collected under approved ethical protocols and allowed to liquefy at RT. Ehtical approval was obtained from the local ethical committee of the medical faculty (University of Duisburg-Essen, file no. 14-5748-BO). The proportions of motile bull and human sperm were increased by a swim-up procedure. Briefly, 1 ml of ejaculate was overlaid with 4 ml HS and incubated 1 h at 37˚C and 5% CO_2. Motile sperm were sampled from the overlaid medium. Aliquots of the preparations were transferred to 20 μm or 100 μm deep chamber slides (Leja, Nieuw-Vennep, Netherlands). All imaging was conducted within 3 h after sample preparation. Mouse and bull sperm were imaged at 20x and human sperm at 40x magnification (referring to the objective lens).

Holographic imaging

The digital holographic microscope DHM™ T-1000 (Lyncée Tec SA, Lausanne, Switzerland) operates in off-axis transmission mode [30, 60]. Its components include a 666 nm laser diode source, 10x/0.3 NA, 20x/0.4 NA and 40x/0.6 NA objectives, and a Basler avA1000-100gm CCD camera (Basler AG, Ahrensburg, Germany). Factory applied calibrations state system resolutions of 3.7466 and 7.4931 pixels/μm using the supplied 20x and 40x objectives. The supplied, proprietary Koala (V6) and open-source Spyder software allow offline processing of the stored holographic images. Koala numerically calculates XY-plane projection images of the specimen at various focal planes [30, 60]. With the aid of custom-designed Spyder scripts, Koala also is able to track the heads of sperm and report X-, Y-, and Z-plane coordinates for the identified trajectories. In some cases the centroid of the head and its associated intensity also were monitored using the Manual Tracking plug-in of the MBF Collection for Image J. Determination of X-, Y-, and Z-plane coordinates for the sperm flagellum required preliminary frame-by-frame tracing of the flagellar image in stacks of reconstructed XY projections using a macro written in the Igor Pro™ (Wavemetrics, Lake Oswego OR, USA) programming environment. A script prepared in Spyder then allowed interrogation of Koala with the flagellar x,y coordinate matrix to obtain the associated Z-plane coordinates. The distance along the flagellum in the XY projections (Dx,y) was determined geometrically from adjacent pairs of x,y coordinates.

Image analysis

Time series of reconstructed XY projection images (8-bit TIFF format, 100 Fps) were maintained in the 800 x 800 pixel format native to the recording camera. Before tracing of the flagellar images in a copy of the time series, auto-adjustments of image contrast and brightness were applied in ImageJ. For tracking of cell trajectories and monitoring of the intensity of the head centroid, the unadjusted time series was cropped to a rectangular field of view that excluded areas outside of the envelope of the cell trajectory. For production of the associated supporting videos, the stack of cropped images received auto-adjustments of image contrast and brightness in ImageJ prior to storage in AVI format using JPEG compression.

Visualization of the sperm flagellum in 4D used a custom-designed Spyder script provided by Lyncée Tec SA. Visualization of sperm swimming trajectories in 4D used the Gizmo function of IgorPro. IgorPro functions also produced Procrustes alignments and visualization of path trajectories in polar coordinates. All experiments were made at room temperature.

Statistics

Numerical results are presented as mean ± s.e.m. with n = number of determinations and N = number of independent experiments.

Supporting information

S1 Movie. Periodic rolling of sperm cells. A free-swimming mouse sperm as seen in a 2.6s time series of reconstructed projections onto the XY-plane (100 Fps, auto-enhanced brightness and contrast), followed by a 2.3s time series from another free swimming sperm. These Avis for the cells from Fig 1A–1D and Fig 2A–2E, illustrate periodic rolling of the cell between RCh to LCh configurations.
(AVI)

S2 Movie. Z-plane excursions of sperm cells. Animation of the passage of smoothed flagelllar Z-plane waves traveling down the flagellum during ~2 beat cycles of the cell from Fig 1. Successive frames add data sampled from every other frame as in Fig 1B. First trace is green, last trace is heavy black, penultimate trace is heavy grey. The time stamp indicates elapsed time.
(AVI)

S3 Movie. 3D projection of sperm flagellum. The sperm flagellum in 4D as shown in an animation of the 3D projection of Fig 1D (blue trace) and its projections onto the XY, XZ, and YZ planes. Sampling 100 Fps. The traveling waves of Z-plane excursions are easily seen.
(AVI)

S4 Movie. Reconstructed trajectory of a sperm cell. An animated rotation of the reconstructed trajectory of the cell of Fig 2D to more clearly show its 3D character. After 2 complete revolutions, the pitch is then adjusted to optimally show the trajectory as viewed facing the approaching cell head-on. Finally, a rolling ball traces the time course of the trajectory, illustrating its CW chirality.
(AVI)

S5 Movie. Animation of the helical trajectory of a sperm cell. Animated trajectory of a free swimming bull sperm during a 2.5 sec sequence. The sequence is shown twice in different perspectives. A green ball locates the head of the sperm and the color code of the trajectory displays the z value. A green helix that follows the path and a projection on a polar coordinate system in the top left corner of the video point out the clockwise chirality of the path.
(AVI)

S6 Movie. Reconstructed DHM projections of bull and human sperm. Shows 100 Fps time series of reconstructed projections onto the XY-plane for 3 sperm. The first segment is for the free-swimming bull sperm of Fig 4A–4E, illustrating periodic rolling of cell around its long axis. The second segment shows another free-swimming bull sperm, the non-rolling sperm of Fig 4F. The third segment shows a free-swimming human sperm, the cell of Fig 5A–5D. Oscillations in intensity of the head indicate periodic rolling of the cell around its long axis.
(AVI)

S1 Fig. Polynomial regression analysis of z-plane excursions. A polynomial regression proves to fit z plane excursion data adequately. The figure shows the first frames of Fig 1B as Z-plane excursions smoothed by a 7th order polynomial. According to Fig 1B, the first frame 0 is green. The data is overlapped with markers that show the unfitted data in their respective color.
(TIF)

Acknowledgments

We are grateful to D. F. Babcock for technical and editorial assistance. We are grateful to A.G. of Wavemetrics, Inc. for help with the Procrustes alignments, to T. Colomb of Lyncée Tec SA for help with Spyder scripts, and to U. Janowitz of Rinder-Union West eG for providing bovine semen. We also thank C.J. Brokaw, California Institute of Technology, C.B. Lindemann of Oakland University and C.L. Asbury, University of Washington for helpful discussions, and J. Szczyrba, Duisburg-Essen University for critical and careful proofreading. Support was provided by DFG WE 2344/9-1 to G.W. We acknowledge support by the Open Access Publication Fund of the University of Duisburg-Essen.

Author Contributions

Conceptualization: Gunther Wennemuth.

Data curation: Michael Muschol, Caroline Wenders, Gunther Wennemuth.

Formal analysis: Michael Muschol, Gunther Wennemuth.

Funding acquisition: Gunther Wennemuth.

Investigation: Michael Muschol, Caroline Wenders, Gunther Wennemuth.

Methodology: Michael Muschol, Gunther Wennemuth.

Project administration: Gunther Wennemuth.

Resources: Gunther Wennemuth.

Software: Michael Muschol.

Supervision: Gunther Wennemuth.

Validation: Michael Muschol, Caroline Wenders, Gunther Wennemuth.

Visualization: Michael Muschol, Caroline Wenders, Gunther Wennemuth.

Writing – original draft: Michael Muschol, Gunther Wennemuth.

Writing – review & editing: Michael Muschol, Caroline Wenders, Gunther Wennemuth.

References

1. Hino T, Muro Y, Tamura-Nakano M, Okabe M, Tateno H, Yanagimachi R. The Behavior and Acrosomal Status of Mouse Spermatozoa In Vitro, and Within the Oviduct During Fertilization after Natural Mating. Biol Reprod. 2016; 95(3):50. https://doi.org/10.1095/biolreprod.116.140400 PMID: 27417908.
2. Ishikawa Y, Usui T, Yamashita M, Kanemori Y, Baba T. Surfing and Swimming of Ejaculated Sperm in the Mouse Oviduct. Biol Reprod. 2016; 94(4):89. https://doi.org/10.1095/biolreprod.115.135418 PMID: 26962118.
3. Miki K, Clapham DE. Rheotaxis guides mammalian sperm. Curr Biol. 2013; 23(6):443–52. https://doi.org/10.1016/j.cub.2013.02.007 PMID: 23453951; PubMed Central PMCID: PMCPMC3607503.
4. Suarez SS, Pacey AA. Sperm transport in the female reproductive tract. Hum Reprod Update. 2006; 12 (1):23–37. https://doi.org/10.1093/humupd/dmi047 PMID: 16272225.
5. Yanagimachi R. Mammalian Fertilization. Raven, New York. 1994:189–317.
6. Babcock DF, Wandernoth PM, Wennemuth G. Episodic rolling and transient attachments create diversity in sperm swimming behavior. BMC Biol. 2014; 12:67. https://doi.org/10.1186/s12915-014-0067-3 PMID: 25182562; PubMed Central PMCID: PMCPMC4354980.
7. Nosrati R, Driouchi A, Yip CM, Sinton D. Two-dimensional slither swimming of sperm within a micrometre of a surface. Nat Commun. 2015; 6:8703. https://doi.org/10.1038/ncomms9703 PMID: 26555792; PubMed Central PMCID: PMCPMC4667638.

8. Brokaw CJ. Thinking about flagellar oscillation. Cell Motil Cytoskeleton. 2009; 66(8):425–36. https://doi.org/10.1002/cm.20313 PMID: 18828155.

9. Brokaw CJ. Computer simulation of flagellar movement X: doublet pair splitting and bend propagation modeled using stochastic dynein kinetics. Cytoskeleton (Hoboken). 2014; 71(4):273–84. https://doi.org/10.1002/cm.21168 PMID: 24574072.

10. Friedrich BM, Riedel-Kruse IH, Howard J, Julicher F. High-precision tracking of sperm swimming fine structure provides strong test of resistive force theory. J Exp Biol. 2010; 213(Pt 8):1226–34. https://doi.org/10.1242/jeb.039800 PMID: 20348333.

11. Lindemann CB, Lesich KA. Flagellar and ciliary beating: the proven and the possible. J Cell Sci. 2010; 123(Pt 4):519–28. https://doi.org/10.1242/jcs.051326 PMID: 20145000.

12. Woolley DM. Flagellar oscillation: a commentary on proposed mechanisms. Biol Rev Camb Philos Soc. 2010; 85(3):453–70. https://doi.org/10.1111/j.1469-185X.2009.00110.x PMID: 20002389.

13. Carlson AE, Hille B, Babcock DF. External Ca2+ acts upstream of adenylyl cyclase SACY in the bicarbonate signaled activation of sperm motility. Dev Biol. 2007; 312(1):183–92. https://doi.org/10.1016/j.ydbio.2007.09.017 PMID: 17950270; PubMed Central PMCID: PMCPMC2259292.

14. Carlson AE, Westenbroek RE, Quill T, Ren D, Clapham DE, Hille B, et al. CatSper1 required for evoked Ca2+ entry and control of flagellar function in sperm. Proc Natl Acad Sci U S A. 2003; 100(25):14864–8. https://doi.org/10.1073/pnas.2536658100 PMID: 14657352; PubMed Central PMCID: PMCPMC299831.

15. Dudiki T, Kadunganattil S, Ferrara JK, Kline DW, Vijayaraghavan S. Changes in Carboxy Methylation and Tyrosine Phosphorylation of Protein Phosphatase PP2A Are Associated with Epididymal Sperm Maturation and Motility. PLoS One. 2015; 10(11):e0141961. https://doi.org/10.1371/journal.pone.0141961 PMID: 26569399; PubMed Central PMCID: PMCPMC4646675.

16. Jansen V, Alvarez L, Balbach M, Strunker T, Hegemann P, Kaupp UB, et al. Controlling fertilization and cAMP signaling in sperm by optogenetics. Elife. 2015; 4. https://doi.org/10.7554/eLife.05161 PMID: 25601414; PubMed Central PMCID: PMCPMC4298566.

17. Nolan MA, Babcock DF, Wennemuth G, Brown W, Burton KA, McKnight GS. Sperm-specific protein kinase A catalytic subunit Calpha2 orchestrates cAMP signaling for male fertility. Proc Natl Acad Sci U S A. 2004; 101(37):13483–8. https://doi.org/10.1073/pnas.0405580101 PMID: 15340140; PubMed Central PMCID: PMCPMC518783.

18. Qi H, Moran MM, Navarro B, Chong JA, Krapivinsky G, Krapivinsky L, et al. All four CatSper ion channel proteins are required for male fertility and sperm cell hyperactivated motility. Proc Natl Acad Sci U S A. 2007; 104(4):1219–23. https://doi.org/10.1073/pnas.0610286104 PMID: 17227845; PubMed Central PMCID: PMCPMC1770895.

19. Wandernoth PM, Mannowetz N, Szczyrba J, Grannemann L, Wolf A, Becker HM, et al. Normal Fertility Requires the Expression of Carbonic Anhydrases II and IV in Sperm. J Biol Chem. 2015; 290(49):29202–16. https://doi.org/10.1074/jbc.M115.698597 PMID: 26487715; PubMed Central PMCID: PMCPMC4705926.

20. Wennemuth G, Westenbroek RE, Xu T, Hille B, Babcock DF. CaV2.2 and CaV2.3 (N- and R-type) Ca2+ channels in depolarization-evoked entry of Ca2+ into mouse sperm. J Biol Chem. 2000; 275(28):21210–7. https://doi.org/10.1074/jbc.M002068200 PMID: 10791962.

21. Xie F, Garcia MA, Carlson AE, Schuh SM, Babcock DF, Jaiswal BS, et al. Soluble adenylyl cyclase (sAC) is indispensable for sperm function and fertilization. Dev Biol. 2006; 296(2):353–62. https://doi.org/10.1016/j.ydbio.2006.05.038 PMID: 16842770.

22. Awata J, Song K, Lin J, King SM, Sanderson MJ, Nicastro D, et al. DRC3 connects the N-DRC to dynein g to regulate flagellar waveform. Mol Biol Cell. 2015; 26(15):2788–800. https://doi.org/10.1091/mbc.E15-01-0018 PMID: 26063732; PubMed Central PMCID: PMCPMC4571338.

23. Heuser T, Barber CF, Lin J, Krell J, Rebesco M, Porter ME, et al. Cryoelectron tomography reveals doublet-specific structures and unique interactions in the I1 dynein. Proc Natl Acad Sci U S A. 2012; 109(30):E2067–76. https://doi.org/10.1073/pnas.1120690109 PMID: 22733763; PubMed Central PMCID: PMCPMC3409752.

24. Ounjai P, Kim KD, Lishko PV, Downing KH. Three-dimensional structure of the bovine sperm connecting piece revealed by electron cryotomography. Biol Reprod. 2012; 87(3):73. https://doi.org/10.1095/biolreprod.112.101980 PMID: 22767409; PubMed Central PMCID: PMCPMC3464910.

25. Satir P, Heuser T, Sale WS. A Structural Basis for How Motile Cilia Beat. Bioscience. 2014; 64(12):1073–83. https://doi.org/10.1093/biosci/biu180 PMID: 26955066; PubMed Central PMCID: PMCPMC4776691.

26. Su TW, Choi I, Feng J, Huang K, McLeod E, Ozcan A. Sperm trajectories form chiral ribbons. Sci Rep. 2013; 3:1664. https://doi.org/10.1038/srep01664 PMID: 23588811; PubMed Central PMCID: PMCPMC3630328.

27. Su TW, Choi I, Feng J, Huang K, Ozcan A. High-throughput analysis of horse sperms' 3D swimming patterns using computational on-chip imaging. Anim Reprod Sci. 2016; 169:45–55. https://doi.org/10.1016/j.anireprosci.2015.12.012 PMID: 26826909.

28. Su TW, Xue L, Ozcan A. High-throughput lensfree 3D tracking of human sperms reveals rare statistics of helical trajectories. Proc Natl Acad Sci U S A. 2012; 109(40):16018–22. https://doi.org/10.1073/pnas.1212506109 PMID: 22988076; PubMed Central PMCID: PMCPMC3479566.

29. Jikeli JF, Alvarez L, Friedrich BM, Wilson LG, Pascal R, Colin R, et al. Sperm navigation along helical paths in 3D chemoattractant landscapes. Nat Commun. 2015; 6:7985. https://doi.org/10.1038/ncomms8985 PMID: 26278469; PubMed Central PMCID: PMCPMC4557273.

30. Colomb T, Pavillon N, Kuhn J, Cuche E, Depeursinge C, Emery Y. Extended depth-of-focus by digital holographic microscopy. Opt Lett. 2010; 35(11):1840–2. https://doi.org/10.1364/OL.35.001840 PMID: 20517434.

31. Cummins JM, Woodall PF. On mammalian sperm dimensions. J Reprod Fertil. 1985; 75(1):153–75. PMID: 4032369.

32. Wennemuth G, Carlson AE, Harper AJ, Babcock DF. Bicarbonate actions on flagellar and Ca2+ -channel responses: initial events in sperm activation. Development. 2003; 130(7):1317–26. PMID: 12588848.

33. Xu G, Wilson KS, Okamoto RJ, Shao JY, Dutcher SK, Bayly PV. Flexural Rigidity and Shear Stiffness of Flagella Estimated from Induced Bends and Counterbends. Biophys J. 2016; 110(12):2759–68. Epub 2016/06/23. https://doi.org/10.1016/j.bpj.2016.05.017 PMID: 27332134; PubMed Central PMCID: PMCPMC4919507.

34. Bukatin A, Kukhtevich I, Stoop N, Dunkel J, Kantsler V. Bimodal rheotactic behavior reflects flagellar beat asymmetry in human sperm cells. Proc Natl Acad Sci U S A. 2015; 112(52):15904–9. https://doi.org/10.1073/pnas.1515159112 PMID: 26655343; PubMed Central PMCID: PMCPMC4703022.

35. Bayly PV, Lewis BL, Kemp PS, Pless RB, Dutcher SK. Efficient spatiotemporal analysis of the flagellar waveform of Chlamydomonas reinhardtii. Cytoskeleton (Hoboken). 2010; 67(1):56–69. Epub 2010/02/20. https://doi.org/10.1002/cm.20424 PMID: 20169530; PubMed Central PMCID: PMCPMC4109274.

36. Kantsler V, Dunkel J, Blayney M, Goldstein RE. Rheotaxis facilitates upstream navigation of mammalian sperm cells. Elife. 2014; 3:e02403. https://doi.org/10.7554/eLife.02403 PMID: 24867640; PubMed Central PMCID: PMC4031982.

37. Klingenberg CP. Size, shape, and form: concepts of allometry in geometric morphometrics. Dev Genes Evol. 2016; 226(3):113–37. https://doi.org/10.1007/s00427-016-0539-2 PMID: 27038023; PubMed Central PMCID: PMCPMC4896994.

38. Gower JC. Generalized Procrustes Analysis. Psychometrika. 1975; 40(1):33–51. https://doi.org/10.1007/Bf02291478 WOS:A1975AC39100002.

39. Rappaz B, Cano E, Colomb T, Kuhn J, Depeursinge C, Simanis V, et al. Noninvasive characterization of the fission yeast cell cycle by monitoring dry mass with digital holographic microscopy. J Biomed Opt. 2009; 14(3):034049. Epub 2009/07/02. https://doi.org/10.1117/1.3147385 PMID: 19566341.

40. Mai A, Weerachatyanukul W, Tomietto M, Wayner DD, Wells G, Balhorn R, et al. Use of atomic force microscopy for morphological and morphometric analyses of acrosome intact and acrosome-reacted human sperm. Mol Reprod Dev. 2002; 63(4):471–9. https://doi.org/10.1002/mrd.10195 PMID: 12412050.

41. Heuser T, Dymek EE, Lin J, Smith EF, Nicastro D. The CSC connects three major axonemal complexes involved in dynein regulation. Mol Biol Cell. 2012; 23(16):3143–55. https://doi.org/10.1091/mbc.E12-05-0357 PMID: 22740634; PubMed Central PMCID: PMCPMC3418309.

42. Inaba K. Sperm flagella: comparative and phylogenetic perspectives of protein components. Mol Hum Reprod. 2011; 17(8):524–38. https://doi.org/10.1093/molehr/gar034 PMID: 21586547.

43. Geyer VF, Sartori P, Friedrich BM, Julicher F, Howard J. Independent Control of the Static and Dynamic Components of the Chlamydomonas Flagellar Beat. Curr Biol. 2016; 26(8):1098–103. https://doi.org/10.1016/j.cub.2016.02.053 PMID: 27040779.

44. Ishijima S. Self-Sustained Oscillatory Sliding Movement of Doublet Microtubules and Flagellar Bend Formation. PLoS One. 2016; 11(2):e0148880. https://doi.org/10.1371/journal.pone.0148880 PMID: 26863204; PubMed Central PMCID: PMCPMC4749303.

45. Lindemann CB, Lesich KA. Functional anatomy of the mammalian sperm flagellum. Cytoskeleton (Hoboken). 2016; 73(11):652–69. https://doi.org/10.1002/cm.21338 PMID: 27712041.

46. Phillips DM. Comparative analysis of mammalian sperm motility. J Cell Biol. 1972; 53(2):561–73. PMID: 5025110; PubMed Central PMCID: PMCPMC2108714.

47. Rikmenspoel R. The tail movement of bull spermatozoa. Observations and model calculations. Biophys J. 1965; 5(4):365–92. https://doi.org/10.1016/S0006-3495(65)86723-6 PMID: 5893155; PubMed Central PMCID: PMCPMC1367881.

48. Ishijima S, Hamaguchi MS, Naruse M, Ishijima SA, Hamaguchi Y. Rotational movement of a spermatozoon around its long axis. J Exp Biol. 1992; 163:15–31. PMID: 1556511.

49. Woolley DM, Osborn IW. Three-dimensional geometry of motile hamster spermatozoa. J Cell Sci. 1984; 67:159–70. PMID: 6746770.

50. Yeung CH, Woolley DM. Three-dimensional bend propagation in hamster sperm models and the direction of roll in free-swimming cells. Cell Motil. 1984; 4(3):215–26. PMID: 6744387.

51. Allen MJ, Rudd RE, McElfresh MW, Balhorn R. Time-dependent measure of a nanoscale force-pulse driven by the axonemal dynein motors in individual live sperm cells. Nanomedicine. 2010; 6(4):510–5. https://doi.org/10.1016/j.nano.2009.12.003 PMID: 20060073.

52. Ishijima S, Hamaguchi Y. Relationship between direction of rolling and yawing of golden hamster and sea urchin spermatozoa. Cell Struct Funct. 1992; 17(5):319–23. PMID: 1473162.

53. Woolley DM. Motility of spermatozoa at surfaces. Reproduction. 2003; 126(2):259–70. PMID: 12887282.

54. Gibbons IR, Shingyoji C, Murakami A, Takahashi K. Spontaneous recovery after experimental manipulation of the plane of beat in sperm flagella. Nature. 1987; 325(6102):351–2. https://doi.org/10.1038/325351a0 PMID: 3808030.

55. Eshel D, Shingyoji C, Yoshimura K, Gibbons BH, Gibbons IR, Takahashi K. Transient behavior of sea urchin sperm flagella following an abrupt change in beat frequency. J Exp Biol. 1990; 152:441–51. PMID: 2230640.

56. Shingyoji C, Katada J, Takahashi K, Gibbons IR. Rotating the plane of imposed vibration can rotate the plane of flagellar beating in sea-urchin sperm without twisting the axoneme. J Cell Sci. 1991; 98 (Pt 2):175–81. PMID: 2055955.

57. Nosrati R, Graham PJ, Liu Q, Sinton D. Predominance of sperm motion in corners. Sci Rep. 2016; 6:26669. https://doi.org/10.1038/srep26669 PMID: 27211846; PubMed Central PMCID: PMCPMC4876399.

58. Lesich KA, de Pinho TG, Dang L, Lindemann CB. Ultrastructural evidence that motility changes caused by variations in ATP, Mg2+, and ADP correlate to conformational changes in reactivated bull sperm axonemes. Cytoskeleton (Hoboken). 2014; 71(11):649–61. https://doi.org/10.1002/cm.21199 PMID: 25430605.

59. Sartori P, Geyer VF, Howard J, Julicher F. Curvature regulation of the ciliary beat through axonemal twist. Phys Rev E. 2016; 94(4–1):042426. Epub 2016/11/15. https://doi.org/10.1103/PhysRevE.94.042426 PMID: 27841522.

60. Rappaz B, Marquet P, Cuche E, Emery Y, Depeursinge C, Magistretti P. Measurement of the integral refractive index and dynamic cell morphometry of living cells with digital holographic microscopy. Opt Express. 2005; 13(23):9361–73. PMID: 19503137.
